# Enhancing the Bioactivity of Bicyclic Peptides Targeted to Grb7-SH2 by Restoring Cell Permeability

**DOI:** 10.3390/biomedicines10051145

**Published:** 2022-05-16

**Authors:** Natasha P. Sturre, Rhys N. Colson, Neelam Shah, Gabrielle M. Watson, Xue Yang, Matthew C. J. Wilce, John T. Price, Jacqueline A. Wilce

**Affiliations:** 1 Department of Biochemistry and Molecular Biology, Biomedicine Discovery Institute, Monash University, Wellington Road, Clayton, VIC 3800, Australia; natasha.sturre@monash.edu (N.P.S.); rhys.colson@monash.edu (R.N.C.); neelam.8.shah@gsk.com (N.S.); watson.g@wehi.edu.au (G.M.W.); xue.yang@monash.edu (X.Y.); matthew.wilce@monash.edu (M.C.J.W.); john.price@vu.edu.au (J.T.P.); 2 Institute for Health and Sport, Victoria University, Melbourne, VIC 8001, Australia

**Keywords:** Grb7, SH2 domain, binding affinity, bicyclic peptide inhibitor, cell permeability, penetratin, breast cancer cell migration

## Abstract

The development of peptide inhibitors against intracellular targets depends upon the dual challenge of achieving a high affinity and specificity for the target and maintaining cellular permeability for biological activity. Previous efforts to develop bicyclic peptides targeted to the Grb7 signalling protein implicated in HER2+ve cancer progression have resulted in improved affinity. However, these same peptides demonstrated a lowered activity due to their decreased ability to penetrate cell membranes. Here, we report the testing of a new series of bicyclic G7 peptides designed to possess improved bioactivity. We discovered that the incorporation of two amino acids (Phe-Pro, Phe-Trp or Phe-Arg) within the bicyclic peptide framework maintains an enhanced binding affinity for the Grb7-SH2 domain compared to that of the first-generation monocyclic peptide G7-18NATE. Structure determination using X-ray crystallography revealed that the mode of binding by the expanded bicyclic G7 peptide is analogous to that of G7-18NATE. Interestingly, while the bicyclic peptide containing Phe-Trp did not display the highest affinity for Grb7-SH2 in the series, it was the most potent inhibitor of HER2+ve SKBR3 breast cancer cell migration when coupled to Penetratin. Together, this demonstrates that peptide flexibility as well as the amino acid tryptophan can play important roles in the uptake of peptides into the cell.

## 1. Introduction

Targeting signalling proteins is an identified strategy against cancers in which specific signalling pathways have become dysregulated. To this end, peptide inhibitors are an attractive option over small molecule inhibitors due to the reliance of signalling pathways on protein–protein interface interactions. Protein–protein interfaces are not effectively disrupted by small molecules but can be targeted by peptides that possess a greater capacity to mimic protein surfaces and to form higher affinity interactions. Peptides have thus been the inspiration for many anti-cancer inhibitor development programs [1,2]. However, while there has been great success in the development of high-affinity and specific peptides and peptidomimetics to protein targets, the problem of delivery remains the greatest obstacle to their implementation [3]. Many peptides are not naturally cell permeable and require conjugation to additional moieties, such as cell-penetrating carrier peptides, to be delivered into the cytosol of the cell. While there is hope that this strategy will provide a viable option for peptide therapeutics development [4,5], there is still much more that needs to be understood about the determinants of peptide cellular uptake. Here, we explore the problem of delivery of a bicyclic peptide targeted to the Grb7 adapter protein by the cell-penetrating peptide Penetratin.

Growth factor receptor-bound protein 7 (Grb7) is an adaptor protein that is important in breast cancer cell migration and proliferation [6,7]. Initially identified due to its co-overexpression with HER2, Grb7 has since been identified as an independent prognostic marker in breast cancer [8,9]. Grb7 mediates interactions between autophosphorylated receptor tyrosine kinase (RTK) and downstream signalling molecules [10]. Specifically, Grb7 promotes the signal transduction of epidermal growth factor receptor (EGFR), HER2, and human epidermal growth factor receptor types 3 and 4 (HER3 and HER4) [11,12,13]. Grb7 has also been demonstrated to promote cell migration through its interactions with focal adhesion kinase (FAK) [7,14,15]. Grb7 primarily interacts with its upstream signalling partners through its Src homology 2 (SH2) domain, leading to Grb7 tyrosine phosphorylation and signal transduction [16,17,18]. It has therefore been hypothesised that inhibiting the Grb7-SH2 domain would inhibit breast cancer cell migration as well as other Grb7-SH2-related signal transduction pathways. Pero et. al. [19] identified a non-phosphorylated peptide titled “G7-18NATE” using phage display. This cyclic peptide is 11 amino acids in length (sequence: WFEGYDNTFPC) and is highly specific to Grb7-SH2 [20]. Through the addition of the cell-penetrating peptide known as Penetratin (hereby referred to as Pen), G7-18NATE-Pen was found to successfully inhibit Grb7-SH2 signal transduction. Specifically G7-18NATE-Pen was found to inhibit breast cancer cell migration and invasion as well as ERK/MAPK and AKT phosphorylation in vitro [7]. In pancreatic mouse models, G7-18NATE-Pen was shown to successfully inhibit the interaction between Grb7 and FAK and, as a result, decrease tumour weight and the number of nodules [21].

This initial success of G7-18NATE led to the development of a series of second-generation Grb7-SH2 inhibitors, with a 130-fold improvement made to the affinity of the interaction with Grb7-SH2 [22,23,24]. These improvements involved the addition of a covalent tether, making the peptide bicyclic, and the removal of two unnecessary amino acids at positions 9 and 10 as well as the incorporation of phosphotyrosine mimetics. This culminated in the development of a nine-amino-acid (sequence: KFEGYDNEC) bicyclic peptide scaffold with the amino acid sequence termed “G7-B7” (or Peptide 5) with a K_D_ of 0.27 µM [22]. Despite G7-B7 possessing the higher affinity to Grb7-SH2 in vivo, it was discovered to be less active than its original counterpart, G7-18NATE, in vitro [24]. While G7-18NATE-Pen was able to effectively reduce the cell migration of breast cancer cells in a wound healing assay, G7-B7-Pen showed no activity. This was found to be correlated to its reduced ability to interact effectively with lipid membranes to enter the cell and exert its inhibitory function upon Grb7 [24]. Despite being conjugated to Penetratin, which is usually very effective at delivering its peptide cargo, the G7-B7 peptide remained membrane-impermeable.

**Figure 1:**
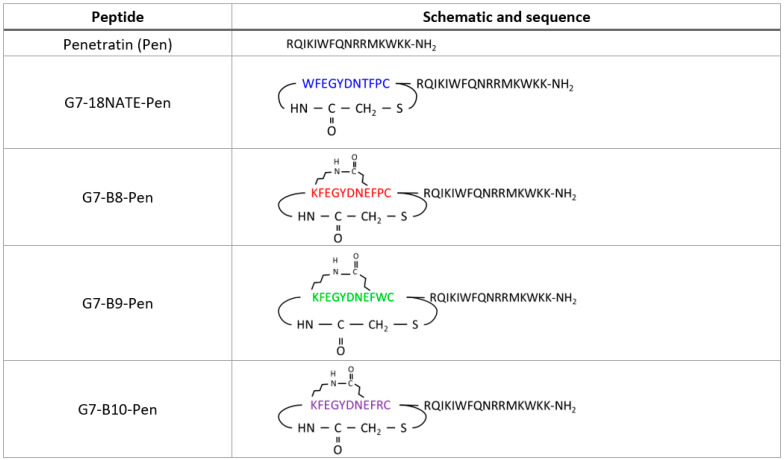
Schematic and peptide sequence of third-generation Grb7-SH2 inhibitors. Shown are schematic representations of the Penetratin-linked peptides used in the current study. The Penetratin amino acid sequence is shown in black single-letter code. The G7 peptide sequences are shown in coloured single-letter code. The thioether linkage that tethers the N-terminal amine to the cysteine side chain and the lactam bridge that tethers Lys1 to Glu8 in the bicyclic peptide are also depicted. Note that binding and structural studies were conducted on G7 peptides synthesized without the Penetratin sequence.

The current study was therefore undertaken to explore the basis for the lost cell permeability and to restore activity to the bicyclic G7-B7 peptide scaffold. It was hypothesised that returning the sequence of G7-B7 to closer to that of G7-18NATE while maintaining the bicyclic constraint could result in increased cell permeability while retaining the affinity for Grb7-SH2. Therefore, a series of third-generation peptides was developed with specific amino acids added to the sequence of G7-B7. The peptides were named G7-B8, G7-B9, and G7-B10 (with sequences as shown in Figure 1). G7-B8 was designed with the premise of making the sequence more similar to that of G7-18NATE. Therefore, the Phe9 and Pro10 that had previously been removed to rigidify the scaffold [23] were re-added to the peptide. G7-B9 and G7-B10 were designed with Trp or Arg replacing Pro10, respectively. These residues were selected, as they are known for their roles in promoting membrane interactions and cell permeability [25]. In particular, Trp was considered a potentially important amino acid residue for the cell permeability of G7-18NATE that was no longer present at position 1 in the bicyclic peptide due to its replacement by Lys to form a lactam linkage. We discovered that all three newly developed peptides possessed enhanced affinities for Grb7-SH2 compared to the original peptide, G7-18NATE. In particular, the G7-B8 peptide showed the highest affinity of the three. The structure determination of the Grb7-SH2/G7-B8 complex using X-ray crystallography revealed that G7-B8 bound Grb7-SH2 through an analogous binding mode to that of G7-18NATE. Interestingly, wound healing assays of Penetratin-conjugated versions of the peptides in HER2+ SKBr3 cell lines revealed that G7-B9, despite possessing the lowest affinity of the G7-bicyclic peptides, was the most effective at inhibiting wound closure. Our study thus reveals the crucial role of the amino acid Trp for the cell permeability of the G7 peptide and illustrates the way in which small changes to peptide cargo can have a big impact on its biological activity.

## 2. Materials and Methods

### 2.1. Peptide Synthesis and Concentration Determination

Peptides were synthesised commercially (Purar Chemicals, Doncaster East, Victoria, Australia). All peptides were purified to greater than 95% purity using RP-HPLC and verified using mass spectrometry. The peptide concentration was determined by absorbance at 280 nm using a NanoDrop 2000/2000c (Thermo Scientific) using calculated extinction coefficients.

### 2.2. Protein Expression and Purification

GST and GST-Grb7-SH2 proteins were expressed and purified as previously described [26]. Briefly, Grb7-SH2 was expressed as a GST fusion, and protein was purified via GST affinity chromatography, followed by size exclusion chromatography.

### 2.3. Surface Plasmon Resonance

SPR experiments were carried out as previously described using the BIAcore T100 [23,26]. BIAcore CM5 series S sensor chips (GE Life Sciences, Paramatta, NSW, Australia) were utilised. Amine coupling was used to immobilise the anti-GST antibody (Abcam, Cambridge, UK) to all flow cells. The immobilisation levels were between 11,000 RU and 7700 RU. As a control, the first flow cell captured only GST. GST-Grb7-SH2 was immobilised on flow cell 2 at close to 2800 RU. Peptides were resuspended in an analysis buffer consisting of 50mM Na_3_PO_4_, 150–300 mM NaCl, and 1 mM DTT (pH 7.4). The peptide was injected over flow cells at 30 μL/min for 60–80 s in triplicates. Double-referenced SPR data were analysed using Scrubber 2.0 (BioLogic Software, Campbell, ACT, Australia) and Prism 9.2.0 (GraphPad Prism v9.0, San Diego, CA, USA).

### 2.4. Crystal Structure Determination

Crystal screening of the Grb7-SH2/B7-B8 complex was performed with sparse screens, including Index and PEG/ION HT (Hampton Research), with Grb7-SH2 at two concentrations (10 mg/mL and 5 mg/mL) and using 300 nL sitting drops. G7-B8 was combined with the Grb7-SH2 domain in a 2:1 molar ratio. Several conditions that gave rise to precipitate and spherulites were observed in the Index screen condition F10. Hand trays set up using the higher 10 mg/mL Grb7-SH2 sample and conditions of 0.2M NaCl, 0.1M BIS TRIS pH 6.6, and 16% PEG3350 gave rise to thin plate crystals. A single crystal was cryoprotected in mother liquor supplemented with 15% (*v*/*v*) glycerol and flash-cooled in liquid nitrogen prior to the collection of diffraction images. The diffraction images were collected at 100 K at the Australian Synchrotron MX2 high-throughput protein crystallography beamline with an ADSC Quantum 315r CCD detector using the BLU-ICE acquisition software [27]. The data were indexed using DIALS [28] and scaled using AIMLESS [29] from the CCP4 suite [30]. The diffraction limit of the data was determined to be to a 2.55 Å resolution. Phases were generated using molecular replacement, using MOLREP [31] from CCP4, with one chain of protein-only Grb7-SH2 as the search model (PDB ID: 5EEQ). A subsequent model refinement was conducted using PHENIX [32], and model building was performed with COOT [33]. The coordinates and data were deposited in the Protein Data Bank (PDB ID: 7MP3).

### 2.5. Cell Culture and Reagents

SKBr3 human breast cancer cell lines were purchased from the American Type Culture Collection (ATCC, Manassas, VA, USA) and were routinely cultured. The SKBr3 cell lines were maintained in McCoy’s 5A medium supplemented with 10% FBS at 37 °C with 5% CO_2_ according to the ATCC guidelines.

### 2.6. Wound Healing Assay

SKBr3 cells were plated in a 24-well plate at 90% confluency and were left to adhere overnight. The cells were serum-starved overnight, and the following day, cells were pre-treated for 2 h with 5 µg/mL mitomycin C (Sigma-Aldrich, St. Louis, MO, USA, M4287) to inhibit proliferation [24]. The medium was replaced with PBS, and a wound was created using a 10 μL pipette tip. The cells were washed twice with PBS to remove cell debris from the wound. The cells were then treated with a 20 μM peptide (control peptide Penetratin, G7-18NATE-Pen, G7-B8-Pen, G7-B9-Pen, or G7-B10-Pen) in complete medium (10% FBS). The complete medium was supplemented with 1 ng/mL EGF to promote migration [6]. The cells were maintained at 37 °C in 5% CO_2_. Wound closure was captured using a Nikon Eclipse TI microscope. An image of the wound was taken every 15 min using NIS-Elements Imaging Software Version 4.20 for 24 h.

The percentage of wound closure was calculated using the following equation:

$$\%\;wound\;closure=\frac{Size\;of\;wound\;at\;time\;0-Size\;of\;wound\;at\;time\;x\;}{Size\;of\;wound\;at\;time\;0}\;$$

## 3. Results

### 3.1. G7 Peptide Binding to Grb7-SH2

**Figure 2:**
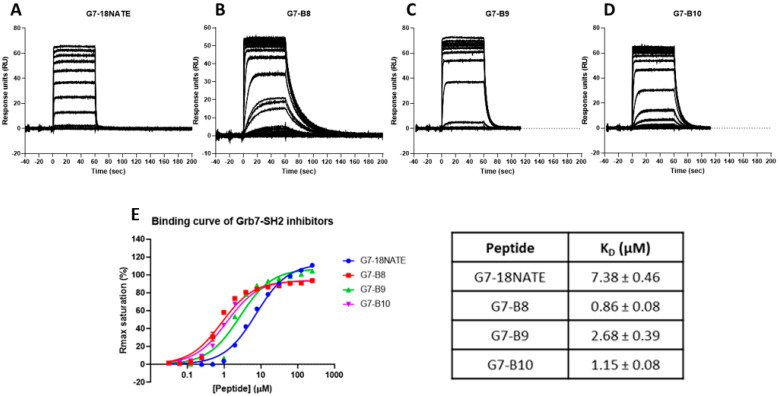
SPR binding analysis of newly developed Grb7-SH2-targeting peptides. Sensograms acquired for (**A**) G7-18NATE, (**B**) G7-B8, (**C**) G7-B9, and (**D**) G7-B10 peptides binding to the Grb7-SH2 domain. Peptide concentrations ranged from 0.015 to 250 μM. (**E**) Binding curves derived for each peptide concentration at steady-state and equilibrium binding constants (K_D_), calculated using a single-site binding model. Errors are SD arising from model.

In order to determine the relative affinities of the newly developed G7 peptides for Grb7-SH2, surface plasmon resonance (SPR) was undertaken using experimental procedures similar to those previously published [22]. Here, it should be noted that the peptides were tested in the absence of the Penetratin tail shown in the schematic (Figure 1). In brief, peptides, at a range of concentrations, flowed over the chip surface to which the Grb7-SH2 domain was tethered, and the response was detected over time. The sensograms revealed that all four peptides readily interacted with Grb7-SH2. The sensogram profile of G7-18NATE was as previously observed [20,26,34]. G7-18NATE quickly reached equilibrium upon injection, and the response readily returned to baseline after the injection period, reflecting a fast dissociation from Grb7-SH2 (Figure 2A). G7-B8 gave rise to a similar sensogram profile to that of G7-18NATE. However, it was observed that G7-B8 had a prolonged dissociation phase, indicative of a higher affinity interaction (Figure 2B). Lower concentrations of G7-B8 did not appear to reach equilibrium. Both G7-B9 and G7-B10 bound Grb7-SH2, and all peptide concentrations reached equilibrium (Figure 2C,D). Interestingly, G7-B9 and G7-B10 also displayed prolonged dissociation phases compared to that of G7-18NATE. However, this was not as pronounced as observed for G7-B8. In order to quantitate the binding affinities, the equilibrium dissociation binding constants (K_D_) for each peptide were calculated using binding curves derived from the sensogram responses at equilibrium (Figure 2E). K_D_ values of 7.83 ± 0.46 µM, 0.86 ± 0.08 µM, 2.68 ± 0.39 µM, and 1.15 ± 0.408 µM were calculated for G7-18NATE, G7-B8, G7-B9, and G7-B10, respectively (Figure 2E). Thus, G7-B8, containing the original Phe-Pro sequence, was revealed to have the highest affinity for Grb7-SH2 compared to the other peptides. These data demonstrate that the addition of two amino acids into the bicyclic scaffold is compatible with binding to Grb7-SH2 and that all three third-generation peptides possess a tighter binding affinity than the original peptide, G7-18NATE.

### 3.2. G7-B8 Binds Grb7-SH2 in a Similar Fashion to G7-18NATE

**Figure 3:**
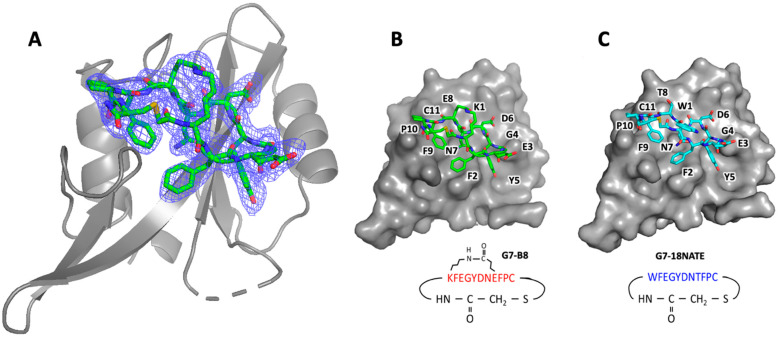
Structure of the G7-B8 peptide bound to the Grb7-SH2 domain compared to that of G7-18NATE. (**A**) The structure of G7-B8 (green stick representation) is shown within the calculated electron density (blue mesh) bound to the Grb7 SH2 domain (grey cartoon representation) (PDB ID: 7MP3). (**B**) The same structure of G7-B8 (green stick representation) is shown bound to the Grb7 SH2 domain (grey surface representation). A schematic representation of the G7-B8 peptide showing the amino acid sequence in single-letter code (red font) is presented underneath. (**C**) The structure of G7-18NATE (blue stick representation) bound to the Grb7-SH2 domain (grey surface representation) is shown from the same orientation for comparison (PDB ID:3PQZ).

In previous studies of G7 bicyclic peptide interactions with the Grb7-SH2 domain, we discovered that the G7-B1 peptide, containing an O-allylserine-based olefin staple group between positions 1 and 8, bound to the Grb7-SH2 domain with a relatively high affinity but in an unexpected orientation [23,35]. Therefore, to assess the mode of binding of G7-B8 to Grb7-SH2, the structure of the Grb7-SH2/G7-B8 complex was determined to a 2.55 Å resolution using X-ray crystallography (Figure 3, Table 1). Interestingly, the complex crystallised with four protein molecules in the asymmetric unit, of which only two Grb7-SH2 domains were formed in complex with the peptide. The other two protein molecules remained as apo-proteins, as similarly observed for G7-18NATE bound to the Grb7-SH2 domain [36]. The mode of G7-B8’s interaction with the Grb7-SH2 domain was strikingly similar to that previously observed for G7-18NATE, as would be expected for this peptide that differs by only two amino acids (Figure 3). The thioether-linked cyclic backbone of G7-B8 made equivalent contacts with the Grb7-SH2 domain surface, including the positioning of Tyr5 in the phosphotyrosine binding pocket, and Phe2 and Phe5 formed planar hydrophobic contacts over the SH2 domain surface. Asn7 formed hydrogen bond interactions with the Grb7-SH2 domain Leu481, which is understood to underlie the Grb7-SH2 domain specificity for the “YXN” motif [17]. Only amino acids 1 and 8 differed in their positioning relative to G7-18NATE due to their substitution with Lys and Glu residues in G7-B8 for the formation of the lactam ring. This linkage can be seen clearly within the G7-B8 electron density (Figure 3A) and reveals that it serves well to support the adopted conformation for the interaction with Grb7-SH2, underlying its enhanced binding affinity.

### 3.3. Impact of G7 Peptides on Migration of HER2+ Breast Cancer Cells

**Figure 4:**
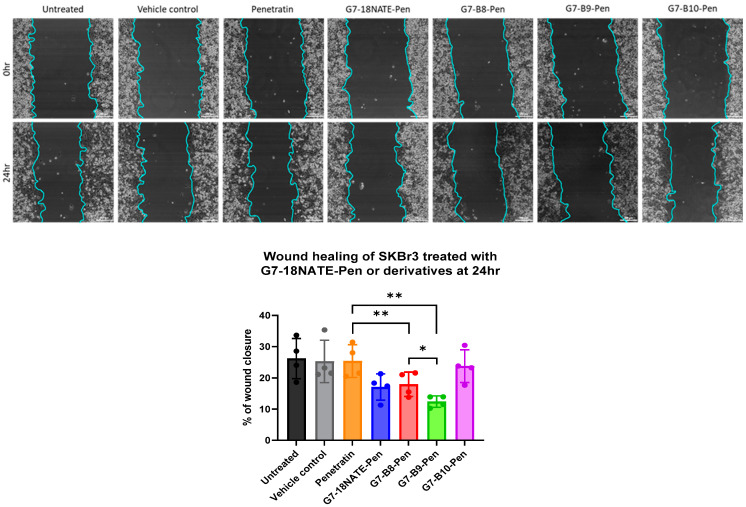
Effects of G7-18NATE-Pen and derivatives upon wound healing of SKBr3 cells. (**top**) Cells were plated as a confluent monolayer, and a wound was created to assess the extent in which the newly developed peptides could inhibit cell migration. The cells were treated with either complete medium (untreated), a vehicle control (sterile PBS), 20 µM control peptide (Penetratin), or 20 µM Grb7-SH2 inhibitor (G7-18NATE-Pen, G7-B8-Pen, G7-B9-Pen, and G7-B10-Pen). Cell migration and wound closure were monitored in real time, and images were captured every 15 min for 24 h. Representative images of wound closure of SKBr3 at 0 h and 24 h. Scale bar is equal to 200 µm. (**bottom**) Percentage of wound closure was calculated over 24 h and paired t-tests were performed without correction. Error bars represent the mean ± SD (n = 4), * = *p* ≤ 0.05, ** = *p* ≤ 0.01.

Following the confirmation that the newly developed peptides bound Grb7-SH2 with a relatively high affinity, their ability to inhibit cell migration was assessed. All peptides were prepared with the C-terminal Penetratin sequence for direct comparison (Figure 1). The peptides were tested for their ability to inhibit the cell migration of HER2+ SKBr3 cells using a wound healing assay. Cells were treated with the G7 peptide or Penetratin alone at a 20 µM concentration for direct comparison with previously reported studies of G7-B7 peptides [22,24]. Over a 24 h period, a similar degree of cell migration was observed for the control cells, the vehicle control (PBS), and Penetratin alone (Figure 4). The G7-18NATE-Pen peptide was found to inhibit cell migration, as previously observed [21,24]. G7-B8-Pen inhibited wound closure, similar to G7-18NATE-Pen. G7-B9-Pen was the most effective at inhibiting wound closure in the SKBr3 cell line. In contrast, G7-B10-Pen did not appear to have any effect upon the wound healing abilities of the SKBr3 cells. Together, these data suggest that the presence of Phe-Trp within G7-B9 successfully restores the peptides’ ability to enter the cell and results in a Grb7-targeted peptide with enhanced biological activity compared to G7-18NATE-Pen.

## 4. Discussion

Grb7 is identified as an independent prognostic marker in breast cancer and is identified as a potential target for the treatment of HER2+ve and TNBC tumours [8,37,38]. The inhibition of this adaptor protein has been demonstrated to reduce cell invasion, migration, and proliferation in several breast cancer cell types [6,7,39]. The discovery has led to the development of a highly specific Grb7-SH2 cyclic binding peptide “G7-18NATE” [19] as well as second-generation bicyclic peptides with improved binding affinities [22,36]. A higher affinity was achieved with the substitution of amino acids Trp1 and Thr8 with lysine and glutamic acid that facilitated the formation of a lactam linker designed to stabilise the bound conformation of the peptide. Peptide rigidity was further enhanced with the removal of amino acids Phe9 and Pro10 [23]. These same changes that gave rise to the enhanced binding affinity of the new bicyclic peptide scaffold, however, also had the effect of reducing the ability of the G7 peptides to enter cells and exert their biological activity, despite conjugation to the cell-penetrating peptide Penetratin [24].

The reason for the lost membrane permeability was potentially due to two factors: First, the increased rigidity of the bicyclic scaffold may have been detrimental to the ability of the peptide to interact with membranes. This could occur, for example, by limiting the formation of intramolecular hydrogen bonds that reduce the desolvation energy penalties during passive diffusion across cell membranes [40]. Second, the replacement of Trp1 to facilitate the formation of the lactam linker between amino acid positions 1 and 8 may have impacted the G7 peptide’s ability to interact with membranes. Tryptophan residues have been identified for their ability to interact with membranes and assist peptide partitioning to just below the lipid headgroups of the lipid bilayer [41]. Both arginine and tryptophan are able to interact with membranes though ion-pair pi interactions at the membrane surface as well as their hydrophobic character, but the presence of tryptophan has been shown to be of particular importance [42].

Thus, it was of interest to determine whether the addition of amino acids that could both restore a degree of peptide flexibility and increase the number of membrane-enhancing amino acids would overall lead to G7 peptides with enhanced activity over the original G7-18NATE. Three peptides were designed with Phe-Pro, Phe-Trp, or Phe-Arg incorporated at positions 9 and 10 of the amino acid sequence. All three G7 peptides were found to bind to the Grb7-SH2 domain with a higher affinity than G7-18NATE. Thus, it can be concluded that the lactam linkage confers a degree of rigidity sufficient to enhance the binding affinity of the G7 peptide. Notably, G7-B8, which is most similar in sequence to G7-18NATE, was found to possess the highest affinity of the three, with a K_D_ of 0.86 µM. This represents an approximately 8-fold increase in affinity over G7-18NATE derived purely via the inclusion of the lactam linkage between positions 1 and 8 in the sequence. This compares favourably with a stapled version of the same sequence, termed G7-B1, that was found to have a K_D_ of 1.5 µM for the Grb7-SH2 domain [23]. This also confirms that amino acid sidechains of Trp1 and Thr8 are not required for interactions directly with the Grb7-SH2 domain.

We were able to successful determine the structure of the G7-B8 peptide in complex with the Grb7-SH2 domain. This was undertaken since, in previous work, the G7-B1 peptide was found to interact with the Grb7-SH2 domain in an unexpected binding mode with the O-allylserine-based olefin staple group forming direct contact with the surface of the Grb7-SH2 domain in place of the Phe9-Pro10 amino acid residues [23]. The G7-B8 peptide, however, bound in the expected orientation, making the same contacts with the surface of the Grb7-SH2 domain as first observed for G7-18NATE [36]. It can thus be concluded that the lactam linkage, unlike the more hydrophobic olefin staple, did not outcompete Phe9-Pro10 to form surface contacts with the Grb7-SH2 domain. Furthermore, it is apparent from the binding affinity measurements that proline is the optimum amino acid at position 10. Neither G7-B9 nor G7-B10 bound Grb7-SH2 with as high an affinity as G7-B8. Thus, proline, either due to its stable turn conformation or the nature of its hydrocarbon sidechain that is able to make hydrophobic contacts with the Grb7-SH2 surface, underlies the higher affinity of the G7-B8 peptide.

In contrast to the relative affinities of the four peptides for the Grb7-SH2 domain (G7-B8 > G7-B10 > G7-B9 > G7-18NATE), the biological activities of the Penetratin-linked G7 peptides revealed a different trend. Here, it should be noted that the current study only utilized the wound healing assay in SKBr3 cells to assess the effect of inhibiting Grb7 on cell migration. Previous studies have explored the activity of Grb7 inhibitor peptides more broadly, including signalling assays, cell proliferation assays, and invasion assays as well as the use of other cell types [24]. While we have found that the biological activity has generally been very well reflected in the wound healing assay in SKBr3 cells, it could nevertheless be useful to verify the biological activity using other assays as well. Despite G7-B9 possessing the lowest affinity for the Grb-SH2 domain, with a K_D_ of 2.68 µM, G7-B9-Pen demonstrated the greatest ability to inhibit SKBr3 cells in a wound healing assay of cell migration. It is unlikely that this is due to a new mode of action of this peptide or a change in peptide stability, though these possibilities cannot be ruled out. It is more likely that, of the G7 peptides, G7-B9 is superior with respect to its ability to enter cells. This peptide differs from G7-B8 and G7-B10 by only one amino acid residue, a tryptophan at position 10. Thus, it suggests that, indeed, tryptophan assists the uptake of the G7 peptide into cells. It was also of interest that the G7-B8-Pen peptide showed equivalent activity to that of G7-18NATE-Pen. While not containing a tryptophan residue, it maintained a sufficient ability to enter cells and exert activity. This is in contrast to the highly rigid G7-B7-Pen peptide tested in a previous study that showed no cellular activity, despite G7-B7 possessing an affinity of K_D_ = 0.27 µM for Grb7-SH2 [22,24]. Thus, in addition to tryptophan, peptide flexibility likely also plays a role in G7 peptide cellular uptake.

## 5. Conclusions

Together, this study highlights that small changes in peptide sequence can impact both the affinity of the peptide for its target and also the cell permeability of the peptide, even when conjugated to a cell-penetrating peptide. We have identified, in the case of the G7 peptides, that extra amino acids at positions 9 and 10 may, in part, contribute to cell permeability by permitting a degree of peptide flexibility. More significantly, however, is that tryptophan, but not arginine was able to enhance cell permeability and therefore the bioactivity to the G7 peptide. The G7-B9-Pen peptide is thus the first Grb7-targetting peptide to display enhanced bioactivity over the originally discovered G7-18NATE-Pen peptide. These peptides both continue to serve a useful role for studies of Grb7 inhibition in cancer cell lines and other tumour models and will help to establish the therapeutic potential of Grb7-SH2 inhibitors.

## Figures and Tables

**Table 1:** X-ray data statistics.

	Grb7-SH2/G7-B8
*Data collection*	
Wavelength (Å)	0.954
Space group	P1
Unit cell dimensions	
a, b, c (Å)	42.16, 53.12, 54.81
α, β, γ (°)	103.98, 102.00, 100.05
Resolution (Å)	42.5–2.55 (2.64–2.55)
^†^ R_merge_ (%)	0.2832 (0.7805)
Wilson B factor	35.42
CC (1/2) (%)	0.912 (0.564)
I/σI	8.33 (1.28)
Unique reflections measured	14071 (1408)
Completeness (%)	98.18% (97.56%)
Multiplicity	4.2 (4.3)
*Refinement*	
R_work_ (%)	0.22 (0.27)
R_free_ (%)	0.26 (0.38)
No. of atoms	
Macromolecules	3193
Ligands	20
Solvent	52
Mean B-factors (Å^2^)	
Macromolecules	40.56
Ligands	37.22
Solvent	35.62
RMSDs	
Bond lengths (Å)	0.002
Bond angles (°)	0.51
Ramachandran plot (%)	
Favoured regions	99.00
Outliers	0.00

† R_merge_ = Σ_hkl_ Σ_i_|I_i_(hkl)-‹I(hkl)›|/Σ_hkl_ Σ_i_I_i_(hkl) where I_i_(hkl) is the ith intensity measurement of reflection hkl, and ‹I(hkl)› is its average. Values given in parentheses are for the high-resolution shell.

## Data Availability

The atomic coordinates and structure factors for the Grb7-SH2/G7-B8 complex were deposited in the RCSB PDB under the accession number PDB: 7MP3.
